# Managing Type 2 Diabetes During the COVID-19 Pandemic: Scoping Review and Qualitative Study Using Systematic Literature Review and Reddit

**DOI:** 10.2196/49073

**Published:** 2024-08-08

**Authors:** Meghan S Nagpal, Niloofar Jalali, Diana Sherifali, Plinio P Morita, Joseph A Cafazzo

**Affiliations:** 1 Institute of Health Policy, Management and Evaluation Dalla Lana School of Public Health University of Toronto Toronto, ON Canada; 2 Centre for Digital Therapeutics Techna Institute University Health Network Toronto, ON Canada; 3 School of Public Health and Health Systems University of Waterloo Waterloo, ON Canada; 4 School of Nursing McMaster University Hamilton, ON Canada; 5 Institute of Biomedical Engineering University of Toronto Toronto, ON Canada; 6 Department of Computer Science University of Toronto Toronto, ON Canada

**Keywords:** type 2 diabetes, social media, patient-generated health data, big data, machine learning, natural language processing, COVID-19, COVID-19 stress syndrome, health behaviors, Reddit, qualitative, analysis, diabetes, scoping review

## Abstract

**Background:**

The COVID-19 pandemic impacted how people accessed health services and likely how they managed chronic conditions such as type 2 diabetes (T2D). Social media forums present a source of qualitative data to understand how adaptation might have occurred from the perspective of the patient.

**Objective:**

Our objective is to understand how the care-seeking behaviors and attitudes of people living with T2D were impacted during the early part of the pandemic by conducting a scoping literature review. A secondary objective is to compare the findings of the scoping review to those presented on a popular social media platform Reddit.

**Methods:**

A scoping review was conducted in 2021. Inclusion criteria were population with T2D, studies are patient-centered, and study objectives are centered around health behaviors, disease management, or mental health outcomes during the COVID-19 pandemic. Exclusion criteria were populations with other noncommunicable diseases, examining COVID-19 as a comorbidity to T2D, clinical treatments for COVID-19 among people living with T2D, genetic expressions of COVID-19 among people living with T2D, gray literature, or studies not published in English. Bias was mitigated by reviewing uncertainties with other authors. Data extracted from the studies were classified into thematic categories. These categories reflect the findings of this study as per our objective. Data from the Reddit forums related to T2D from March 2020 to early March 2021 were downloaded, and support vector machines were used to classify if a post was published in the context of the pandemic. Latent Dirichlet allocation topic modeling was performed to gather topics of discussion specific to the COVID-19 pandemic.

**Results:**

A total of 26 studies conducted between February and September 2020, consisting of 13,673 participants, were included in this scoping literature review. The studies were qualitative and relied mostly on qualitative data from surveys or questionnaires. Themes found from the literature review were “poorer glycemic control,” “increased consumption of unhealthy foods,” “decreased physical activity,” “inability to access medical appointments,” and “increased stress and anxiety.” Findings from latent Dirichlet allocation topic modeling of Reddit forums were “Coping With Poor Mental Health,” “Accessing Doctor & Medications and Controlling Blood Glucose,” “Changing Food Habits During Pandemic,” “Impact of Stress on Blood Glucose Levels,” “Changing Status of Employment & Insurance,” and “Risk of COVID Complications.”

**Conclusions:**

Topics of discussion gauged from the Reddit forums provide a holistic perspective of the impact of the pandemic on people living with T2D, which were found to be comparable to the findings of the literature review. The study was limited by only having 1 reviewer for the literature review, but biases were mitigated by consulting authors when there were uncertainties. Qualitative analysis of Reddit forms can supplement traditional qualitative studies of the behaviors of people living with T2D.

## Introduction

### Background

Type 2 diabetes (T2D) is characterized by the body’s resistance or insufficient production of insulin. Research suggests that the risks of further complications for people living with T2D can be mitigated through proper self-management [1]. Treatment protocol for proper management of T2D includes glycemic control, weight management, adequate nutrition, regular physical activity, reducing sedentary behaviors, and taking prescribed medications [2].

### COVID-19 and Managing T2D

With the emergence of the COVID-19 pandemic, beginning in March 2020, social distancing measures included business closures, remote school and work measures, prohibition of large crowds, limited socialization outside the household, and increased reliance on digital health care delivery. As a result of these changes and fear of the unknown, stress and anxiety were resulting manifestations [3]. People living with diabetes are already at increased risk for serious complications from COVID-19 due to already being immunocompromised and because the virus may thrive in an environment of high blood glucose [4]. A scoping review conducted in 2023 by Li et al [5] revealed that diabetes prevalence increased among those with severe COVID-19, accounting for 16.8% of deaths. Therefore, it was vital that those living with T2D took extra precautions to avoid the virus. However, proper management of T2D requires healthy lifestyle behaviors, which were likely impacted by the lifestyle changes that occurred during lockdowns, in addition to the exacerbated risk of attaining severe COVID-19 symptoms.

### Study Objective and Rationale

Considering that proper management of T2D requires healthy behaviors and that the implications of the COVID-19 pandemic were disruptive in people’s daily lives worldwide, this study aimed to consolidate the literature of studies that examined the health behaviors and attitudes of people living T2D during the first year of the COVID-19 pandemic and to compare the themes gauged from the scoping review to topics of discussion on Reddit forums among people living with T2D during the same period. Social media is a form of patient-generated health data where users can discuss with their peers how they manage T2D through sharing diet, food, symptoms, research, and recipes while obtaining peer support [6]. It also presents a public data source to gauge sentiment and topics of discussion during the initial lockdown period. Our objective was to examine if data from social media, in this case Reddit, provided insights that were similar to findings from the literature review.

## Methods

### Scoping Review

A scoping review was conducted following the framework of Arksey and O’Malley [7] using the following steps: (1) identifying the research question, (2) identifying relevant studies, (3) study selection, (4) charting the data, and (5) summarizing and reporting the results [8].

### Search Strategy

Searches were conducted in 3 databases (PubMed, Scopus, and CINAHL) from January 2020 to May 2021 (Textbox 1). Using the keywords identified, relevant studies were identified using the inclusion and exclusion criteria from title and abstract to full-text screening. For studies included in the data charting phase, reference lists were scanned for any additional relevant studies. However, these searches did not produce any additional results.

**Search terms for the scoping review.**

*Diabetes* AND (manag* OR behave* OR *mental* OR *stress* OR *anxiety* OR *depression*) AND *(COVID* OR *coronavirus* OR *pandemic*)

### Study Selection

Following the Arksey and O’Malley [7] framework, papers were reviewed in 3 iterations. In the first iteration, abstracts were scanned and selected using the eligibility criteria below. In the second iteration, the full text was scanned using the same eligibility criteria to select papers. Finally, in the third iteration, data were extracted and charted, and studies were excluded if they did not meet the eligibility criteria. Eligibility was determined based on the criteria below and for a paper to be included, all inclusion criteria needed to be met, and not have met any exclusion criteria. Only 1 reviewer (MSN) performed the initial study screening and assessment, but uncertainties about inclusion criteria were addressed to the other authors and solved through discussion to make the final decision for study eligibility.

The inclusion criteria are (1) the population of focus must include people living with T2D, (2) the findings of the study are patient-centered, and (3) the objectives of the study are to gauge changes in health behaviors, disease management, or mental health outcomes during the COVID-19 pandemic.

The exclusion criteria are (1) people living with other noncommunicable diseases, not as a comorbidity with T2D (an exception to this criterion was made if the population consisted of people with type 1 diabetes or gestational diabetes), (2) the study examines COVID-19 as a comorbidity to T2D, (3) clinical treatments or delivery of care for COVID-19 among people living with T2D, (4) genetic expressions of COVID-19 among people living with T2D, (5) case studies, commentary, review papers, or gray literature (ie, letters to editor, editorials, blogs, and newspapers), or (6) studies not published in English.

### Charting and Extracting Data

To guide data extraction, parameters were created that included the country of study, the time in which the study was conducted, the study sample size, and the main findings. Findings were directly extracted and quoted from the paper and the remaining data parameters were interpreted through analysis from examining the paper. Given the limitations of the study, only 1 reviewer (MSN) was able to conduct the extraction, but clarity was taken from other authors when there were areas of uncertainty.

### Synthesis of Data

The extracted results from the study were examined and were given numerical codes for thematic analysis. Thematic analysis was conducted by the primary reviewer (MSN) and other authors were consulted in the case of uncertainty or discrepancies. Themes were categorized to summarize the studies by their main findings to answer the research question.

### Examination of Reddit Data

#### Data Collection

For this study, 3 communities on Reddit were examined: r/type2diabetes, r/diabetes_t2, and r/diabetes [9-11]. From the r/diabetes [11] community, only posts that were tagged with the “flair” and “type 2 diabetes” were examined. The former 2 communities are exclusively for people living with T2D, while the latter was only examined if it was tagged as T2D. While there is no way to guarantee that patients living with type 1 diabetes were excluded from this data set, it was reasonable to assume that the discussions in our data set only pertained to T2D, given that they were posted or tagged in communities for people living with T2D.

Reddit was the chosen data source because there were readily available open-source application programming interfaces (APIs) to harness the data through Python scripts. Additionally, because Reddit communities are divided by different interest groups, such as diabetes, it ensured that the data source mostly consisted of the population of interest. Finally, Reddit’s terms and conditions did not forbid the use of data for research purposes and was chosen for that reason [12].

#### Classification of Posts

A year’s worth of data were examined, a total of 48,988 posts from March 2020 to March 2021. Within the data set, terms related to the COVID-19 pandemic were manually searched for using search features on Excel (Microsoft Corp) by MSN. These terms included *COVID*, *coronavirus*, *pandemic*, *social distancing*, *lockdown*, *quarantine*, *toilet paper*, *unemploy*, *unemployed*, *work from home*, *working from home*, *telehealth*, *vaccine*, *sanitizer*, and *mask*.

Posts that contained those terms in the text body were manually evaluated for context and labeled as “covid” or “noncovid.” In total, 9803 posts were manually classified by MSN and verified by NJ, with 2065 labeled as “covid” and 7738 labeled as “noncovid” and subsequently classified with the support vector machines. An additional 818 posts that were published in the context of the COVID-19 pandemic were identified, bringing the total number of pandemic-specific posts to 2883. The remainder of unlabeled posts published in the identified pandemic period were labeled as “noncovid.”

#### Data Analysis

**Figure 1:**
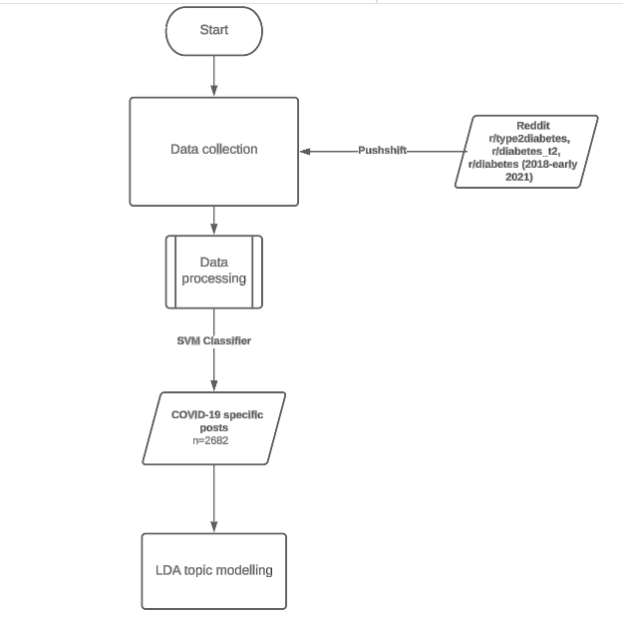
Collecting and processing of Reddit data. LDA: latent Dirichlet allocation; SVM: support vector machine.

The latent Dirichlet allocation (LDA) topic modeling algorithm [13,14] with the MALLET (Machine Learning for Language Toolkit) package [15] was used to obtain topics of discussion by obtaining clusters of words belonging to a single topic (Figure 1). This unsupervised algorithm was chosen as there was no precedent of topics that were being detected, and thus, there were no data to train a supervised algorithm. After classifying the posts, the entire data set was reprocessed, with 2682 being specific to the COVID-19 pandemic, and topic modeling was performed. A value of *k*, the number of topics, was determined by evaluating the coherence scores outputted by the model for each value of *k* and by manually evaluating the distinction between topics for various *k* values.

#### Sentiment Analysis

Sentiment analysis (SA) with the VADER (Valence Aware Dictionary for Sentiment Reasoning) [16] algorithm was performed to understand the subjective emotions, or sentiment, associated with each post. Valence scores of sentiment are calculated on both polarity (if the text is negative or positive) and intensity (how positive or negative a text is), with a normalized compound score, returned between –1 and +1. Thresholds for classifying a text as per VADER are as follows [16]: positive: compound score ≥+0.05; neutral: compound score between –0.05 and +0.05; and negative: compound score ≤–0.05.

### Ethical Considerations

As per the University of Toronto’s research ethics guides exemptions in section 1, ethical approval was not obtained for this study because it was assumed that Reddit is a public data source and it is assumed that there is no reasonable expectation of privacy [17]. Moreover, there was no direct interaction between the researcher and the participants, and hence, the researchers did not believe that ethical approval was necessary, as per section 2 [17]. The data used for our qualitative analysis was scraped directly from Reddit and it was assumed to be a public data source. As users do not need a form of authentication to view Reddit forums, it was assumed that the users who posted to them did so with the knowledge that they would be displayed publicly. Data were obtained through a data dump from the Pushshift API, and more than 100 published research studies have already harnessed Reddit data with Pushshift [18]. We do not believe that using this API was a violation of Reddit’s API terms of use [19]. However, in a systematic analysis of 727 manuscripts that used Reddit as a data source, only 15% mentioned any form of ethical review [20]. We do acknowledge that there is debate on the ethics of using Reddit data for academic research purposes but nowhere on Reddit’s terms and conditions prohibit the use of data for research purposes [12]. Hence, as of the time of research, by omission of information about using data for research purposes, it was assumed that it was within Reddit’s terms and conditions. While the username of the post authors was obtained, they were assumed to be pseudonyms of the user and not their actual names. However, we do acknowledge that some users may have integrated their real names into their usernames, but in this study, the usernames were not analyzed to confirm so. Reddit also does not provide identifiable characteristics of individual users, such as their name, gender, or geographical location. However, we do acknowledge that some users may put identifiable information within their text. For this study, this identifiable information was not harnessed or analyzed.

## Results

### Findings From Scoping Review

**Figure 2:**
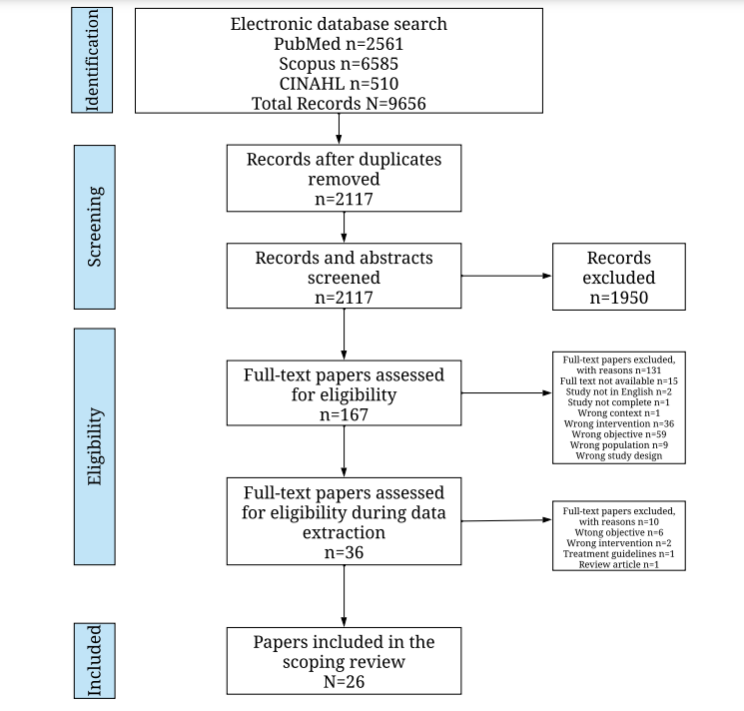
PRISMA (Preferred Reporting Items for Systematic Reviews and Meta-Analyses) diagram from the scoping review.

A total of 9656 papers were identified from the 3 databases. Of these 9656 papers, 7539 (78.1%) papers were duplicates and were subsequently removed. The abstracts of 2117 papers were screened and the full texts of 167 (7.9%) of those papers were scanned to evaluate if they met the inclusion criteria. Of those papers, 36 (1.7%) papers were included for data extraction. Finally, after close examination from data extraction, of the 36 papers, 26 (72.2%) papers were included as part of this scoping review as they met the inclusion criteria. Figure 2 summarizes the process.

### Study Characteristics

A total of 26 studies published as of May 2021 were included in this review. With respect to data collection, of the 26 studies, 21 (80%) studies used surveys or questionnaires, 3 (11%) studies used interviews, 2 (8%) studies analyzed blood glucose readings, and 1 (4%) study used hospital consultations. The studies took place between February and September 2020 and included a sample size of 13,673 people living with diabetes. The geographical representation includes 7 (26%) studies from India, 3 (11%) studies from Japan, 2 (8%) studies from China, 2 (8%) studies from Denmark, 2 (8%) studies from Brazil, 2 (8%) studies from the Netherlands, 1 (4%) study from Turkey, 1 (4%) study from Spain, 1 (4%) study from Germany, 1 (4%) study from Arab Gulf, 1 (4%) study from Mexico, 1 (4%) study from Singapore, 1 (4%) study from Pakistan, and 1 (4%) study from the United Kingdom.

### Findings From Thematic Analysis

The main themes found from the literature review include poor glycemic control, increased consumption of unhealthy foods, reduction in physical activity, and inability to access medical appointments. A detailed summary of all included papers is included in Multimedia Appendix 1.

### Findings From Analysis of Reddit Data

The LDA topic modeling algorithm was performed on 2883 posts published between February 28, 2020, and February 28, 2021.

### Findings From LDA Topic Modeling

Table 1 summarizes the topics and associated words for COVID-19–related posts.

**Table 1:** Topics and associated words for COVID-19–related posts.

Topic number	Topic name	Words
Topic 1	Coping With Poor Mental Health	feel, day, week, work, weight, lose, walk, bit, symptom, bad, lot, gym, night, happen, end, great, covid, felt, ill, diagnosis
Topic 2	Accessing Doctor & Medications and Controlling Blood Glucose	doctor, test, ac, year, insulin, month, metformin, diagnose, low, glucose, diet, week, med, exercise, change, level, stop, reading, check, medication
Topic 3	Changing Food Habits During Pandemic	eat, food, carbs, lot, meal, low, carb, diet, hard, hour, thing, water, keto, fast, cut, easy, glucose, add, stuff, rice
Topic 4	Impact of Stress on Blood Glucose Levels	blood, glucose, high, time, stress, number, long, exercise, bg, body, problem, morning, normal, control, sleep, make, level, effect, change, kind
Topic 5	Changing Status of Employment & Insurance	work, home, hospital, today, time, year, talk, give, advice, wait, insurance, visit, guess, state, order, meter, live, strip, situation, friend
Topic 6	Risk of COVID Complications	covid, type, health, care, risk, sick, control, issue, virus, disease, diabetic, question, pandemic, mask, hand, case, infection, wear, patient, home

### Findings From Sentiment Analysis

Impact of Stress on Blood Glucose Levels and Coping With Poor Mental Health had average compound scores that fell in the threshold of being classified as neutral (odds ratio [OR] 0.0252, 95% CI –0.0344 to 0.0849 and OR 0.0492, 95% CI –0.0121 to 0.1105, respectively). Risk of COVID Complications, Accessing Doctor & Medications and Controlling Blood Glucose, and Changing Status of Employment & Insurance were ranked next from lowest to highest with average compound scores of OR 0.0876 (95% CI 0.0322-0.1430), OR 0.1457 (95% CI 0.0879-0.2035), and OR 0.1748 (95% CI 0.1263-0.2232), respectively, and finally, Changing Food Habits During Pandemic had the highest average compound score of 0.2544 (95% CI 0.1965-0.3123).

## Discussion

### Principal Results

Our analysis revealed that people living with T2D were negatively impacted by the pandemic mentally and were negatively impacted by how they managed their chronic disease. Our literature review found that people living with T2D were negatively impacted by the pandemic by having poorer glycemic control, poorer lifestyle behaviors, their inability to access medical appointments, and increased stress and anxiety. Our analysis of Reddit data found similar themes, with additional emphasis on the economic impacts of the pandemic among people living with T2D.

Our literature review found that the COVID-19 pandemic impacted people’s lives through poorer glycemic control, increased consumption of unhealthy foods, decreases in physical activity, inability to access medical appointments, and increased stress and anxiety toward the impact of the lockdown and fear of being exposed to the coronavirus. Topic modeling from data on 3 Reddit forums for people living with T2D found the following topics: Coping With Poor Mental Health, Accessing Doctor & Medications and Controlling Blood Glucose, Changing Food Habits During Pandemic, Impact of Stress on Blood Glucose Levels, Changing Status of Employment & Insurance, and Risk of COVID Complications. The additional finding of employment as a topic of discussion on Reddit forums suggests that digital discussion presents a holistic perspective of diabetes management that considers the person’s life as a whole when managing their disease.

Furthermore, the majority of the 26 studies included in our literature review mostly relied on surveys and interviews to obtain their data. Surveys and interviews are often time-consuming processes. However, analyzing data from forums such as Reddit, using machine learning algorithms such as topic modeling and SA, can be a quicker method to obtain a broad range of themes and sentiments from a large volume of participants when performing qualitative research. We do not suggest that qualitative analysis from digital forums could replace traditional qualitative research, but can rather supplement it as our study demonstrates that the results from our analysis are comparable to results from traditional qualitative studies of people living with T2D.

### Glycemic Control

As glycemic control is a major component of the self-management of T2D, it was expected that this would be a major theme found both in our literature review and in our analysis of Reddit data. While psychological stress is subjective among individuals, few studies have demonstrated that psychological stressors have been linked to hyperglycemia [21-23]. Considering that the major theme of our findings suggested that the pandemic was a stressor for people living with T2D, it was hypothesized that this stress would have an impact on glycemic control. From our Reddit analysis, the topic Impact of Stress on Blood Glucose Levels had the lowest sentiment score of OR 0.0252 (95% CI –0.0344 to 0.0849), suggesting that there was increased anxiety toward managing blood glucose levels.

Our literature review further reiterated that people living with T2D experienced increased blood glucose or increased hemoglobin A_1c_ (HbA_1c_) levels during the pandemic [24-28]. While 1 study did correlate higher HbA_1c_ with increased levels of stress during the pandemic [29], our literature review also attributed poorer glycemic control as a result of reduced blood glucose monitoring and reduced medical visits being reasons for this [24,30-35]. This sentiment was also reflected in our analysis of Reddit data. The topic Accessing Doctor & Medications and Controlling Blood Glucose considered that reduced access to medication and health care providers impacts blood glucose levels, and while reduced health care visits were a factor in reduced glycemic control, the Reddit analysis additionally considered that losing employment was also a stressor for people living with T2D and also resulted in lost insurance benefits that reduced doctor visits and medication access.

### Lifestyle Management

As T2D is managed by lifestyle behaviors, they were nonetheless a significant theme of discussion found in both our literature review and our analysis of T2D Reddit communities. Our literature review revealed that lifestyle behaviors among people living with T2D were impacted by increased consumption of unhealthy foods and reduced physical activity [24-27,30,33,36-38]. As a result of these behavior changes, participants reported changes in body weight [27,37].

These findings were supported by our analysis of Reddit as Changing Food Habits During Pandemic was a topic of discussion. Interestingly, this topic was associated with the highest average sentiment (OR 0.2544, 95% CI 0.1965-0.3123). While the literature suggests that dietary changes were attributed to stress during the pandemic [33], the positive sentiment score of the Reddit posts may be an indication that increased unhealthy food consumption may have been a coping mechanism associated with positive emotions through the stressful time and that there was a sense of camaraderie and bonding among peers through this coping mechanism.

Considering physical activity, in the topic Coping With Poor Mental Health, the terms “gym” and “walk” were included. This may suggest that stress was related to gym closures in the initial months of the pandemic and people relying on walking as a means of physical activity. The literature review supports that gym closures and having fewer opportunities to walk due to teleworking and closures of businesses are attributed to reducing physical activity [25,27]. Moreover, the literature review attributes changes in exercise behaviors as a result of pandemic-related stress [38], and hence, it was fitting that the topic modeling algorithm pooled terms related to mental health with terms related to physical activity.

### Access to Diabetes Care

During the COVID-19 pandemic, accessing care was perceived as a significant barrier to managing T2D as more clinical visits were done through telehealth as a means to protect patients and health care providers from exposure to the coronavirus. However, not all people living with T2D had access to telehealth care, particularly those living in rural communities [25]. Moreover, health care providers were called to aid in treating patients infected with COVID-19 [25], resulting in people living with T2D being unable to see their regular health care provider or being treated by health care providers who were not experienced in managing T2D [39]. Our literature review revealed that people living with T2D had difficulty managing their blood glucose levels and felt depression as a result of missed medical appointments.

Among our analysis of Reddit data, Access to Doctor & Controlling Blood Glucose was identified as a topic of discussion among people living with T2D, with an average sentiment score (OR) of 0.1457 (95% CI 0.0879-0.2035). Further examination of Reddit data revealed that while barriers to accessing health care providers existed, another barrier was presented through the fear of acquiring COVID-19 infection and avoiding hospitalization in potentially dangerous situations. The fear of acquiring COVID-19 infection was also reflected in our literature review in a general sense. Further research in the years ahead would need to examine the impact of the lessening of in-person health care visits among people living with T2D.

### Mental Health

With the COVID-19 pandemic being disruptive to personal lives worldwide, many people experienced elevated stress and anxiety. The mental health impact of this pandemic is expected to be long-term due to the extreme measures that were necessary to prevent the spread of the virus and the resulting economic implications [40]. Our literature review revealed that people living with T2D were no exception to the stressors of the pandemic which included social isolation [28,29,41] and financial stress [28,29]. However, stressors that were specific to people living with T2D included missing medical appointments [42], being unable to access medications and supplies to manage diabetes [42,43], and managing their disease [29]. Additionally, people living with T2D were anxious about being exposed to COVID-19 as they felt that they were more vulnerable to serious complications or death [26,31,41-44].

Comparing these findings from our literature review to our analysis of Reddit data found Coping With Poor Mental Health, Impact of Stress on Blood Glucose Levels, and Risk of COVID Complications as topics of discussion. Overall, these topics were associated with lower sentiment scores (OR 0.0492, 95% CI –0.0121 to 0.1105; OR 0.0252, 95% CI –0.0344 to 0.0849; and OR 0.0876, 95% CI 0.0322 to 0.1430; respectively). Specifically, within the Reddit analysis, users acknowledge that increased stress during the pandemic impacted their glycemic control but users also felt that they would be helpless if they experienced diabetes-related complications as hospitalization would put them at risk of acquiring a COVID-19 infection. Moreover, as supported in the literature review, people living with T2D were generally afraid of being exposed to the virus with their increased risk state. While our study demonstrated that people living with T2D were using peer support as a means to cope with the stressors of the pandemic, it also demonstrates that users were negatively impacted by the psychological stressors of the pandemic.

### Impact to Employment

Efforts to curb the virus resulted in many employers worldwide requiring their employees to work from home [45], which resulted in changes in work-life balance and mental health issues with the inability to interact with others outside the household [46,47]. Moreover, as businesses shut down during the pandemic to curb the spread of the virus, the pandemic resulted in the loss of employment for many workers. The unemployment rate reached 14.1% in the United States in April 2020, the highest since data collection began in 1948 [48]. Additionally, only 60.2% of the labor force participated in April 2020, the lowest participation observed since the 1970s [48]. Unemployment already poses the issue of loss of income and standard of living and decreased sense of self-purpose [49], potentially impacting health behaviors due to increased stress. However, many people unemployed were also impacted by changes to their health insurance because of their job loss [50,51].

While Kishimoto et al [25] suggested that teleworking resulted in reduced physical activity among people living with T2D, there was little discussion about the financial and employment implications of the pandemic among people living with T2D. However, Changing Status of Employment & Insurance was a topic of discussion among Reddit users on T2D forums. From the Reddit discussions, it could be inferred that a loss of insurance posed a barrier for people living with T2D to get medication and blood glucose meters and strips, affecting glycemic control. Clinicians must consider how one’s employment status affects people living with T2D, especially when they are additionally posed with a barrier to accessing clinical care, and outline treatment options in these situations.

### Comparison to Prior Work

This study builds upon previous studies that have harnessed data from weblogs and social media websites to understand diabetes behaviors and the sentiment associated with the texts. People living with T2D use social media and digital forums to discuss their condition and related information among their peers [52]. These themes of discussion include diet, food, symptoms, research, recipes, and news [52-55]. This study uncovered similar themes of discussion through analysis of Reddit forums, with the added context of the COVID-19 pandemic, given that this study was conducted in 2021, as the pandemic was ravaging globally. However, no other study at the time compared the findings of social media analysis to the findings from traditional qualitative studies. Our study suggests that social media can be a supplemental data source when performing clinical qualitative analysis.

### Comparison of Literature Review to Reddit Analysis

Our literature review summarized 26 studies conducted on 13,673 people living with diabetes to understand how the pandemic impacted and how they managed their diseases. Our analysis of Reddit data used support vector machines to classify Reddit posts written from 2020 to early 2021, published in the context of the pandemic from 1263 distinct authors. Most of the data obtained by researchers of the studies included in the literature review were through surveys and interviews, while the data obtained in the Reddit analysis used APIs to scrape data that were posted by Reddit users and analyzed using LDA topic modeling and VADER SA. As displayed in Textbox 2, the topics gauged from both studies were comparable to one another, with the Reddit analysis gauging an additional topic of Changing Status of Employment & Insurance. This additional finding suggests that discussions on Reddit offer insight from a holistic perspective that considers aspects of a person’s life in the context of their disease, beyond treating symptoms. Moreover, analyses from Reddit forums can be less time-consuming than conducting long surveys or interviews and can collect data from a larger volume of users than from a single study.

**Comparison of topics found in the literature review to topics found in the Reddit discussion.**

**Literature review topics**

- Increased Consumption of Unhealthy Foods
- Decreased Physical Activity
- Inability to Access Medical Appointments
- Anxiety Toward Lockdown
- Fear of COVID Exposure

**Reddit topics**

- Changing Food Habits During Pandemic
- Impact of Stress on Blood Glucose Levels
- Accessing Doctor & Medications and Controlling Blood Glucose
- Coping With Poor Mental Health
- Risk of COVID Complications
- Changing Status of Employment & Insurance

### Limitations

Due to the staffing limitations and reliability of remote work during the pandemic, only 1 reviewer, MSN, was able to conduct the study search and study data extraction. However, any uncertainties were addressed to DS and JAC to mitigate any risk of bias.

Topic modeling and SA were performed on the Reddit posts but the posts were not thoroughly examined for context, and hence, the authors cannot comment on the quality of the discussions posted on the forum. We cannot confirm that the users who published the posts were all people living with T2D. We only examined the 3 Reddit communities mentioned in the study and no other subreddits about the coronavirus or mental health. Furthermore, we had no information about the demographics of the users of the diabetes forums of Reddit and assumed that the demographics were similar to the demographics of all Reddit users. Under this assumption, there could be a sampling bias in our Reddit data as Reddit users are mostly male and fall into the age demographic of 18-49 years [56,57]. Geographically, the United States has the largest number of Reddit users, while other users mostly reside in English-speaking, higher-income countries [58].

### Conclusions

The findings from the literature review included topics of glycemic control, lifestyle management, access to diabetes care, and the impact on mental health among people living with T2D. However, an examination of Reddit data revealed an additional theme of employment being impacted during the pandemic, affecting diabetes lifestyle behaviors. Moreover, Reddit presented a large sample size of participants. Therefore, social media presents an opportunity to holistically observe the behaviors of those managing chronic diseases.

## Appendix

Multimedia Appendix 1 Findings from scoping review.

Multimedia Appendix 2 PRISMA checklist.

## Abbreviations

API: application programming interface
HbA1c: hemoglobin A1c
LDA: latent Dirichlet allocation
MALLET: Machine Learning for Language Toolkit
OR: odds ratio
SA: sentiment analysis
T2D: type 2 diabetes
VADER: Valence Aware Dictionary for Sentiment Reasoning

## Data Availability

The data sets generated during or analyzed during this study are available from the corresponding author upon reasonable request.
