# Supplementary material for: Managing Type 2 Diabetes During the COVID-19 Pandemic: Scoping Review and Qualitative Study Using Systematic Literature Review and Reddit
Source: Interact J Med Res. 2024 Aug 8;13:e49073. doi: 10.2196/49073 (PMC11342012; doi:10.2196/49073)
Supplement: Multimedia Appendix 1 [file ijmr_v13i1e49073_app1.docx]

| Title | Authors | Country | Time Period | Sample Size | Study Tool | Findings |
| --- | --- | --- | --- | --- | --- | --- |
| A cross sectional study reveals severe disruption in glycemic control in people with diabetes during and after lockdown in India | Khader MA, Jabeen T, Namoju R. | India | Jun 10 - July 15, 2020 | 1582 | Online questionnaire | "The frequency of clinical visits during the COVID-19 pandemic were reduced in 87.28% of participants. 92.45% of participants were able to monitor their blood glucose levels (BGLs) in which 78.42% (49.35%, 20.91%, and 8.16%) participants experienced an increase in BGL (mild, moderate, and severe respectively). Only 47.41% of participants possessed the digital glucometer at home. 69.07% of participants reported a decrease in physical activity while 46.88% reported an increase in food intake. 80.06% of participants were able to buy all medicines and 29.80% were gone for virtual consultations while 87.81% reported that they didn’t have access to healthcare services. Overall, 89.47% participants experienced disruption in therapy. A highly significant correlation (r = 0.89, p = 0.0145) was found between increasing age and reporting of higher BGLs." |
| Barriers to Self-Management of Type 2 Diabetes During COVID-19 Medical Isolation: A Qualitative Study | Shi C, Zhu H, Liu J, Zhou J, Tang W. | Wuhan, China | Feb 20 - Mar 4, 2020 | 12 | Semi-structured Telephone interviews | "Perceived barriers to diabetes self-management described by diabetes patients indicated a lack of environmental resources and support strategies to meet their needs. Efforts to remove barriers are important in assisting patients with diabetes to improve their quality of life and health outcomes." |
| Behavioral changes in patients with diabetes during the COVID-19 pandemic | Kishimoto M, Ishikawa T, Odawara M. | Japan | April 1 - Jun 13, 2020 | 168 | Regular hospital consultations | "Among 168 patients, 26 (15.5%) gained > 2 kg; HbA1c levels were elevated or decreased by > 0.2% compared to that at the last visit in 57 and 51 patients (Groups D and I), respectively. Group D patients were affected to a larger extent by changes in commuting (transition to teleworking) and closures of sport gyms than Group I patients. Increased snacks, sweets, total diet, and alcohol intake could have contributed to worsening of glucose control in Group D, whereas a healthy diet and less alcohol intake could have led to better glucose control in Group I." |
| Changes in Sexual Functions and Alexithymia Levels of Patients with Type 2 Diabetes During the COVID-19 Pandemic | Dincer B, Yıldırım Ayaz E, Oğuz A. | Istanbul, Turkey | May 21 - July 5, 2020 | 162 | Data were collected using the Information Form, Toronto Alexithymia Scale, Hospital Anxiety and Depression Scale | "For 83.3% of the participants, there was a decrease in sexual functioning after diabetes, 69.8% after the COVID-19 pandemic, and 67.2% due to both conditions." |
| Coping with diabetes during the COVID - 19 lockdown in India: Results of an online pilot survey | Nachimuthu S, Vijayalakshmi R, Sudha M, Viswanathan V. | India | April 1 - April 15, 2020 | 100 | Online surveys | "92% of the participants had Type 2 diabetes. Only 28% of the participants were checking their blood glucose levels regularly. 80% of the participants mentioned that they were following regular exercise and diet control during the lockdown period. 40% of the participants were anxious about the Covid infection." |
| Covid-19 lockdown and changes of the dietary pattern and physical activity habits in a cohort of patients with type 2 diabetes mellitus | Ruiz-Roso, M.B., Knott-Torcal, C., Matilla-Escalante, D.C., Garcimartín, A., Sampedro-Nuñez, M.A., Dávalos, A., Marazuela, M. | Madrid, Spain | April 8 - May 20, 2020 | 72 | Food Frequency Questionnaire (FFQ), Physical Activity Questionnaire (IPAQ), Food Craving Questionnaire-State (FCQ-S) and Food Craving Questionnaire-Trait (FCQ-T) were used. | "It seems that during lockdown, families tried to ameliorate their dietary habits, as for example, they had more time to cook more elaborate meals and increased their vegetable consumption, but despite this, the overall quality of the diet did not improve. They also showed an increase in glucosey foods and snacks intake, probably due to emotions such as boredom of staying at home all day or the stress caused by the pandemic. In fact, we found an association between levels of foods cravings and snack consumption. Apart from that, this study found a high percentage of physical inactivity before the COVID-19 lockdown, which was exacerbated during home confinement." |
| COVID-19-Related Fear, Risk Perception, and Safety Behavior in Individuals with Diabetes | Musche V, Kohler H, Bäuerle A, Schweda A, Weismüller B, Fink M, Schadendorf T, Robitzsch A, Dörrie N, Tan S, Teufel M, Skoda EM. | Germany | April 9 - Jun 3, 2020 | 253 with diabetes and 253 matched controls | Participants completed an anonymous survey including demographics, depressive symptoms (PHQ-2), generalized anxiety (GAD-7), COVID-19-related fear, risk perception, and safety behavior. (3) | "Patients with diabetes showed no elevated generalized anxiety or depressive symptoms. However, they reported higher COVID-19-related fear and more adherent and dysfunctional safety behavior compared to controls." |
| Determinants of mental health outcomes among people with and without diabetes during the COVID-19 outbreak in the Arab Gulf Region | Al-Sofiani ME, Albunyan S, Alguwaihes AM, Kalyani RR, Golden SH, Alfadda A. | Arab Gulf region | April 21 - May 5, 2020 | 568 with diabetes, 1598 without diabetes | "A cross-sectional questionnaire-based study collecting demographic and mental health data. Depression and anxiety were assessed using the 9-item Patient Health Questionnaire and the 7-item Generalized Anxiety Disorder scale, respectively." | "The prevalence of depression and anxiety symptoms were 61% and 45%, in people with diabetes (PWD) and 62% and 44%, respectively, in people without diabetes. PWD who have had their diabetes visit canceled by the clinic were more likely to report depression and anxiety symptoms than those without diabetes (odds ratio [95% confidence interval]: 1.37 [1.02, 1.84] and 1.37 [1.04, 1.80], for depression and anxiety; respectively). Fear of acquiring the coronavirus infection; running out of diabetes medications; or requiring hospitalization for hypoglycemia, hyperglycemia, or diabetic ketoacidosis; and lack of telecommunication with HCPs were all associated with significantly higher odds of having depression and anxiety symptoms among PWD." |
| Diabetes and COVID-19: psychosocial consequences of the COVID-19 pandemic in people with diabetes in Denmark-what characterizes people with high levels of COVID-19-related worries? | Joensen LE, Madsen KP, Holm L, Nielsen KA, Rod MH, Petersen AA, Rod NH, Willaing I. | Denmark | n.d. | 2430 | Questionnaire | "People with diabetes have COVID-19-specific worries related to their diabetes. More than half were worried about being overly affected due to diabetes if infected with COVID-19, about one-third about being characterized as a risk group due to diabetes and not being able to manage diabetes if infected. Logistic regressions showed that being female, having type 1 diabetes, diabetes complications and diabetes distress, feeling isolated and lonely, and having changed diabetes behaviours were associated with being more worried about COVID-19 and diabetes." |
| Effect in self-care behavior and difficulties in coping with diabetes during the COVID-19 pandemic | SILVA-TINOCO, RUBÉN; GONZÁLEZ-CANTÚ, ARNULFO; DE LA TORRE-SALDAÑA, VIRIDIANA; GUZMÁN-OLVERA, EILEEN; CUATECONTZI-XOCHITIOTZI, TERESA; CASTILLO-MARTÍNEZ, LILIA; ROMERO-IBARGUENGOITIA, MARIA ELENA; NAHUACATL-LÓPEZ, ARELI; CASTILLO-GALINDO, CARMEN; OREA-TEJEDA, ARTURO; SERNA-ALVARADO, JAVIER; LEÓN-GARCÍA, ENRIQUE; OCHOA-MORENO, JORGE | Mexico City, Mexico | April 20 - May 29, 2020 | 212 | Questionnaire | "Self-reported difficulties for coping with T2D were documented and included difficulties in self-care behaviors related to: diet (41.6%), physical activity/exercise (40.5%), glucose monitoring (23.7%), getting pharmacological treatment (22.9%), pharmacological treatment adherence (19%), and home-confinement adherence (11.7%). In the group of patients with a recent previous-lockdown evaluation, global self-care activity decreased from 5.15±0.9 to 4.49±1.02 days/week, p-value<0.001. Patients with T2D reported difficulties in coping with T2D and decreased diabetes self-care activities during the COVID-19 lockdown in Mexico City. Diabetes self-care has an inverse relationship with the presence of difficulties for coping with diabetes." |
| Effect of coronavirus disease 2019 pandemic on the lifestyle and glycemic control in patients with type 2 diabetes: a cross-section and retrospective cohort study | Munekawa C, Hosomi Y, Hashimoto Y, Okamura T, Takahashi F, Kawano R, Nakajima H, Osaka T, Okada H, Majima S, Senmaru T, Nakanishi N, Ushigome E, Hamaguchi M, Yamazaki M, Fukui M. | Japan | April 16 - May 1, 2020 | 203 | Questionnaire | "Increased levels of stress and decreased exercise levels were reported in approximately 40% and >50%. During the COVID-19 pandemic. There was a negative correlation between stress and exercise (r = –0.285, p < 0.001) and a positive correlation between stress and prepared food intake (r = 0.193, p = 0.009). Decreased exercise levels (r = –0.33, p < 0.001) and increased snack consumption (r = 0.24, p = 0.002) were associated with increased body weight. Furthermore, increased total diet intake (r = 0.16, p = 0.031) was associated with increased HbA1c levels." |
| Effects of COVID-19 lockdown on type 2 diabetes, lifestyle and psychosocial health: A hospital-based cross-sectional survey from South India | Sankar P, Ahmed WN, Mariam Koshy V, Jacob R, Sasidharan S. | Southern India | "during 4 weeks ranging from the third week of May to the third week of June 2020" | 110 | "The pre- and post-lockdown data of 110 adults with T2D who were under regular follow up was collected by direct interview during their visit to the diabetes clinic." | "The overall physical activity and dietary adherence remained unchanged in more than 80% of the participants. There was increased consumption of vegetables (80.9%), fruits (42.7%), and decreased unhealthy snacking (63%). 90% of them had access to medications. No significant change was noted in the mean HbA1c and body weight before and after lockdown. Most of them (99%) watched television and 73.6% of them spent time with their family members. Those with mental stress and poor sleep had unhealthy dietary habits. Poor glycemic control was seen in those with less physical activity and an unhealthy diet.' |
| Effects of nationwide lockdown during COVID-19 epidemic on lifestyle and other medical issues of patients with type 2 diabetes in north India | Ghosh A, Arora B, Gupta R, Anoop S, Misra A. | Northern India | “We started interviews on May 10, 2020 and finished this task in 7 days’ time” | 150 | Phone Interview | "Carbohydrate consumption and frequency of snacking increased in 21% and 23% patients, respectively. Interestingly, 27% patients reported an increase in consumption of fruits. Exercise duration was reduced in 42% and weight gain occurred in 19% patients. Frequency of doing self-monitoring of blood glucose (SMBG) was decreased in 23% patients. 'Mental stress' of any kind was reported in 87% patients. Availability of medicines and insulin was uninterrupted in 91% patients. Knowledge about telemedicine was present in 69% and majority (92%) of these patients preferred video consultation." |
| Factors associated with psychic symptomatology in diabetics during the COVID-19 pandemic Fatores associados à sintomatologia psíquica em diabéticos durante a pandemia da COVID-19 | Souza, G.F.A., Praciano, G.A.F., Neto, O.D.C.F., Paiva, M.C., de Jesus, R.P.F.S., Cordeiro, A.L.N., Souza, G.A., Silva Junior, J.R., Souza, A.S.R. | Brazil | April 24 and May 3, 2020 | 162 | Questionnaire | "Frequency of 37.7%, 43.3% and 45.1% were found for some symptom of stress, anxiety and depression, respectively. The factors associated with sign and symptoms of severe / extreme psychic disorders were: not having religion (stress, anxiety and depression); be at graduation (stress and anxiety); history of anxiety and depression (anxiety and depression); not practicing or decreasing physical exercises and starting, increased or continue taking sleeping medications (stress); history of contact with a suspected case of COVID-19, absence or decreased leisure (anxiety); female gender, absence of a partner, decreased family income and work or study (depression)." |
| Impact of COVID-19 and partial lockdown on access to care, self-management and psychological well-being among people with diabetes: A cross-sectional study | Yeoh E, Tan SG, Lee YS, Tan HH, Low YY, Lim SC, Sum CF, Tavintharan S, Wee HL. | Singapore | June and October 2020 | 301 respondents, 68.4% have Type 2 diabetes | Online surveys | "Respondents reported less frequent engagement in physical activity (38%), checking of blood pressure (29%) and blood glucose (22%)." |
| Impact of lockdown on self-care management among patients with type 2 Diabetes Mellitus residing in Lucknow city, India – A cross-sectional study | Tiwari, A., Kumar, D., Ansari, M.S., Chaubey, S.K., Gupta, N.R., Agarwal, V., Chandra, K.P., Pande, A.R., Awasthi, R., Gupta, M., Chowdhary, S. | Lucknow, India | June 2020 | 1406 | Questionnaire | "About 27% were under stress, 14.7% and 30.8% reported a change in dietary and sleep pattern. About 83% could not consult a doctor and as a result 13% stopped taking medications. Patients with stress slept less, observed a change in dietary patterns and had uncontrolled blood glucose levels (P = 0.0001). On the contrary those without stress spent time with family and were occupied with a hobby (P = 0.0001). Those with controlled blood glucose levels exercised more and had normal sleep patterns." |
| Increased stress, weight gain and less exercise in relation to glycemic control in people with type 1 and type 2 diabetes during the COVID-19 pandemic | Ruissen MM, Regeer H, Landstra CP, Schroijen M, Jazet I, Nijhoff MF, Pijl H, Ballieux BEPB, Dekkers O, Huisman SD, de Koning EJP. | Netherlands | 8 – 11 weeks after the lockdown on March 15, 2020 | 435 people (type 1 diabetes n=280, type 2 diabetes n=155) | Questionnaire, HbA1c Analysis | "An increase in perceived stress and anxiety, weight gain and less exercise but no deterioration of glycemic control occurs in both people with relatively well-controlled type 1 and type 2 diabetes during short-term lockdown measures. As perceived stress showed to be associated with glycemic control, this provides opportunities for healthcare professionals to put more emphasis on psychological aspects during diabetes care consultations." |
| Lifestyle changes as a result of COVID-19 containment measures: Bodyweight and glycemic control in patients with diabetes in the Japanese declaration of a state of emergency | Tanaka N, Hamamoto Y, Kurotobi Y, Yamasaki Y, Nakatani S, Matsubara M, Haraguchi T, Yamaguchi Y, Izumi K, Fujita Y, Kuwata H, Hyo T, Yamada Y, Kurose T, Seino Y. | Japan | June 8 - 18, 2020 | 463 | Questionnaire | "After stratification by age 65 years, binary logistic regression analysis showed that increased frequency of snack eating increased bodyweight (odds ratio 1.709, P = 0.007) and glycated hemoglobin (odds ratio 1.420, P = 0.025) in the younger group, whereas in the older patients, reduced walking activities resulted in weight gain (odds ratio 0.726, P = 0.010). In conclusion, changes in eating behavior and physical activity increased bodyweight and reduced glycemic control among diabetes patients, but by different processes depending on age under the coronavirus disease 2019 containment measures in Japan." |
| Mental health in the era of COVID-19: prevalence of psychiatric disorders in a cohort of patients with type 1 and type 2 diabetes during the social distancing | Alessi J, de Oliveira GB, Franco DW, Brino do Amaral B, Becker AS, Knijnik CP, Kobe GL, de Carvalho TR, Telo GH, Schaan BD, Telo GH. | Brazil | n.d. | type 1 (n = 52) and type 2 diabetes (n = 68) | "The primary outcome was the prevalence of minor psychiatric disorders, assessed by survey (SRQ-20). Secondary outcomes included the prevalence of diabetes related emotional distress, eating and sleeping disorders, all assessed by validated surveys at the moment of the study." | "93% of patients showed signs of current mental suffering based on the surveys measured. Almost 43% of patients showed evidence of significant psychological distress, with a significant greater tendency in patients with type 2 diabetes. The presence of diabetes related emotional distress was found in 29.2% of patients; eating disorders in 75.8%; and moderate/severe sleeping disorders in 77.5%." |
| Observational study on Effect of Lock Down due to COVID 19 on glycemic control in patients with Diabetes: Experience from Central India | Khare J, Jindal S. | Central India | Duration of study was 3 weeks during the first phase lock down period. | 407 | "Patients who met the...criteria were included and advised for SMBG (self-monitoring of blood glucose) for minimum 2 readings which included Fasting Blood Glucose (FBG) and Post Prandial Blood Glucose (PPBG)." | "(39.16%) patients reported worsening of hyperglycemia and requiring addition of medications for control of blood glucose and 3 (2.09%) patients reported hypoglycemic events and medications were stepped down. Psychological stress was most common factor worsening of hyperglycemia followed by change in diet and exercise." |
| Perceived risk, behavior changes and Health-related outcomes during COVID-19 pandemic: Findings among adults with and without diabetes in China | Yan, A.F., Sun, X., Zheng, J., Mi, B., Zuo, H., Ruan, G., Hussain, A., Wang, Y., Shi, Z. | China | April 25 and early May 11, 2020 | 9,016 total participants, 585 reported having diagnosed diabetes and 8,431 had no diabetes. | Surveys | "During the COVID-19 pandemic, participants with diabetes were more likely to experience food and drug shortages and to increase their physical activity, compared to their counterparts. Among diabetic respondents, a high proportion of current smokers (74.1%) and drinkers (68.5%) reported increased amounts of smoking and drinking. People with diabetes were 11% less likely to report excellent or very good health. Having 150 min/week physical activity was positively associated with excellent or very good health (prevalence ratio, PR = 1.14, 95%CI 1.11-1.16)." |
| Psychological factors associated with changes in physical activity in Dutch people with type 2 diabetes under societal lockdown: A cross-sectional study | Regeer, H., Nieuwenhuijse, E.A., Vos, R.C., Kiefte-de Jong, J.C., van Empelen, P., de Koning, E.J.P., Bilo, H.J.G., Huisman, S.D. | Netherlands | First week of May 2020 | 536 | Questionnaire | "47% reported no change in PA, 27% became less active and 26% became more active during societal lockdown. Participants who were more likely to become less active were participants who experienced more stress (OR: 2.27; 95% CI 1.25-4.13) or less stress (OR: 2.20; 95% CI 1.03-4.71). Participants who were more likely to become more active were participants who experienced more stress (OR: 2.31; 95% CI 1.25, 4.26). Participants with higher emotional well-being (OR: 0.98; 95% CI 0.97, 0.99) were less likely to become less active than to report no change in PA." |
| Psychometric Analysis for fear of COVID-19 Scale (FCV-19S) and its association with depression in patients with diabetes: A cross sectional study from a Tertiary Care Centre in Karachi, Pakistan | Basit KA, Zafar AB, Fawwad A, Waris N, Shaheen F, Basit A. | Pakistan | August to September 2020 | 380 | "Baseline demographic details were obtained from hospital management system of BIDE. Forward-backward translation method was used to translate the existing Fear scale (FCV–19S). Symptoms of depressive disorder were assessed through Patient Health Questionnaire (PHQ9)." | "Total of 380 participants with mean age 51.93 ± 12.03 years contributed in the study. Three factors loading and item correlation of fear COVID-19 explained 96% of total variance having unidimensional Cronbach’s alpha of 0.881. All demographic indicators that showed significance in univariate model were included in multivariate model. Females had more fear for COVID-19 compared to males (OR = 1.73, 95% CI (1.15-2.6)), whereas current smokers had also showed 4 times more fear than non-smokers (OR = 4.19, 95% CI (1.18-14.83). Depression assessed by PHQ9 showed maximum fear of COVID-19 in participants with moderate depression." |
| Psychosocial health in people with diabetes during the first three months of the COVID-19 pandemic in Denmark | Madsen KP, Willaing I, Rod NH, Varga TV, Joensen LE. | Denmark | March 19 to June 25, 2020 | 1366 | Surveys | "COVID-19 worries, feelings of social isolation, psychological distress, anxiety and general loneliness had all improved at Q6 compared to Q1 (p < 0.001). In general, improvements in psychosocial health started after the first reopening phase (April 15); however, general loneliness increased up to the first reopening phase (p ≤ 0.001) before decreasing, and quality of life decreased up to the first reopening phase (p = 0.002), with no improvements to follow. Subgroup analyses revealed that women had larger decreases in feelings of social isolation (p < 0.001) and in psychological distress (p = 0.035) and increases in quality of life (p < 0.001), between Q1 and Q6, compared to men." |
| Self care practices and psychological distress among diabetic patients in Manipur during COVID-19: A scenario from the North East | Bala R, Srivastava A, Potsangbam T, Anal L, Ningthoujam GD. | India | Aug 3 to Aug 28, 2020 | 108 | "A convenient sampling method was used to recruit subjects from a representative clinical sample using validated scales like the Summary of Diabetes Self-Care Activities (SDSCA) and Kessler Psychological Distress Scale (K10)." | "The everyday healthy eating plan was followed by 76.85% (N = 83) subjects and daily physical activity for at least 30 min performed by 50% (54) subjects. Only 12.04% (13) subjects tested their blood glucose and 6.48% (7) respondents checked their feet daily. There was no significant difference found between the SDSCA and psychological distress based on socio-demographic variables.  Participants in this study typically reported a good level of self-care behavior particularly for diet followed by exercise whereas the self-care behavior was not adequate for foot care and blood-glucose testing. People were not too anxious about COVID-19. This study highlighted the fact that people with diabetes should monitor their blood glucose levels more often along with their foot care." |
| The early impact of the COVID-19 pandemic on adults with type 1 or type 2 diabetes: A national cohort study | Fisher L, Polonsky W, Asuni A, Jolly Y, Hessler D. | United Kingdom | Early April 2020 | 763 (T1D) and 619 (T2D) | Surveys | "There were widespread increases in general and diabetes-related stress and social isolation, and negative effects on disease management. About 25% reported increases in highs, lows, and glucose variability in both groups." |
